# Comparison of Efficacy and Safety of Rivaroxaban, Heparin, and Enoxaparin in Preventing Thrombosis in Gynaecologic Oncology Surgeries: Thromboprophylaxis in Gyn-oncology Surgery

**DOI:** 10.31661/gmj.v14i.3957

**Published:** 2025-11-04

**Authors:** Elham Saffarieh, Shaghayegh Pazoki, Setare Nassiri

**Affiliations:** ^1^ Abnormal Uterine Bleeding Research Center, Semnan University of Medical Science, Semnan, Iran; ^2^ Medical of School, Semnan University of Medical Sciences, Semnan, Iran; ^3^ Endometriosis Research Center, Iran University of Medical Sciences, Tehran, Iran

**Keywords:** Venous Thromboembolism, Anticoagulants, Heparin, Enoxaparin, Rivaroxaban, Gynecologic Surgical Procedures, Randomized Controlled Trial

## Abstract

**Background:**

Background: Background: Venous thromboembolism (VTE) is a frequent and potentially serious complication following gynecologic oncology surgeries. Anticoagulants such as heparin, enoxaparin, and rivaroxaban are commonly used for thromboprophylaxis; however, their comparative efficacy and safety remain uncertain in this patient population.

**Materials and Methods:**

Materials and Methods: This pilot randomized controlled trial included 85 patients undergoing gynecologic oncology surgery, randomly assigned to receive enoxaparin (n=25), heparin (n=30), or rivaroxaban (n=30). Randomization was performed using block randomization (block size=3) with allocation concealment and double blinding of patients and outcome assessors. The trial was registered in the Iranian Registry of Clinical Trials (IRCT20151020024625N19) and approved by the Ethics Committee of Semnan University of Medical Sciences (IR.SEMUMS.REC.1402.223). Baseline data included age, BMI, cancer type, surgical procedure, and history of vascular events. Outcomes comprised transfusion requirement, dyspnea, chest pain, peripheral edema, lower limb pain, bleeding, infection, hematoma, recovery, and mortality. Data analysis was performed using SPSS v.22 (IBM Corp., Armonk, NY, USA).

**Results:**

Results: Fourteen patients (16.5%) required intraoperative transfusion, with a significantly higher rate in the rivaroxaban group (33.3%) compared to enoxaparin (8.0%) and heparin (6.7%) (P=0.010). Peripheral edema was also more common with rivaroxaban (16.7%) than with heparin (3.3%) or enoxaparin (0%) (P=0.046). Other outcomes showed no significant between-group differences (all P0.05).

**Conclusion:**

Conclusions: Rivaroxaban use was linked to increased intraoperative transfusion and short-term edema compared to heparin and enoxaparin. Larger multicenter trials are warranted to confirm these preliminary safety and efficacy findings.

## Introduction

Thrombosis is a very important and fatal complication after surgery, generally occurring in the form of deep vein thrombosis (DVT) or pulmonary embolism (PE) [1][2]. In patients undergoing major gynaecologic surgery, in the absence of thromboprophylaxis, the prevalence of DVT ranges from 15% to 40% [3]. Venous thromboembolism (VTE) is one of the main causes of mortality after gynaecologic and obstetric surgeries [1] In general, the risk of VTE in cancer patients is five to six times higher than in non-cancer patients [2][3]. VTE is an independent prognostic factor for mortality and the second leading cause of death in cancer patients [4][5]. Also, asymptomatic DVT strongly increases the risk of PE [6].

Since most deaths associated with PE occur within 30 minutes of the onset, the time for therapeutic intervention is very limited and it is necessary to identify those at high risk of VTE and to implement effective thromboprophylaxis to minimize mortality in these patients [7]. Despite the advances made in recent years, venous thromboembolism (VTE) still accounts for a high percentage of mortality. Also, cancer increases the risk of VTE 4-7 times, making it the second leading cause of death in these patients [8].

Therefore, patients undergoing surgical intervention for gynaecologic cancer are at high risk of VTE due to both risk factors. One method of preventing thrombosis is the use of anticoagulant drugs such as rivaroxaban, heparin, and enoxaparin [9]. Heparin in combination with antithrombin III prevents clot formation by inactivating factor Xa and inhibiting prothrombin conversion [9][10][11]. Enoxaparin is also a low molecular weight heparin that binds to and activates antithrombin III, thereby inhibiting factors Xa and IIa [10]. In fact, the main effect of this class of drugs is on factor Xa inhibition, with little effect on thrombin (IIa) and clotting time [12]. On the other hand, rivaroxaban is an oral anticoagulant (NOAC). It is the first direct oral factor Xa inhibitor, a small molecule oxazolidinone derivative that binds directly and reversibly to factor Xa through S1 and S4 receptors, and competitively inhibits factor Xa [13][14].

Unlike heparin and enoxaparin, rivaroxaban inhibits both free and clot-bound factors and inhibits prothrombinase activity, thereby prolonging clotting time [15].

Given the importance of thrombosis in patients undergoing surgery, the present study was conducted to compare the efficacy and safety of rivaroxaban, heparin, and enoxaparin in preventing thrombosis in gynaecologic oncology surgeries.

## Materials and Methods

### Study Design and Setting

This study was designed as a single-center pilot randomized controlled trial (RCT) conducted at Hospital, affiliated with Semnan University of Medical Sciences, Iran, The trial was registered in the Iranian Registry of Clinical Trials (IRCT20151020024625N19; https://www.irct.ir/trial/24625) and approved by the Ethics Committee of Semnan University of Medical Sciences (IR.SEMUMS.REC.1402.223). Written informed consent was obtained from all participants prior to enrollment.

### Participants

Eligible patients were women scheduled for gynecologic oncology surgeries, including staging hysterectomy or cytoreductive surgery, with histologically confirmed ovarian, endometrial, or uterine sarcoma. Exclusion criteria included contraindications to anticoagulation, severe renal or hepatic dysfunction, or refusal to participate.

### Sample Size Consideration

As a pilot RCT, the target sample size was pragmatically set at 30 patients per group (total=90), consistent with recommendations for pilot studies. This number was intended to provide preliminary effect estimates for transfusion requirements and complication rates to guide future definitive trials. During the study, 5 patients were excluded, resulting in 85 patients available for final analysis (25 enoxaparin, 30 heparin, 30 rivaroxaban).

### Randomization and Blinding

Patients were randomly allocated into three groups (enoxaparin, heparin, rivaroxaban) using a computer-generated block randomization sequence (block size=3). Allocation concealment was ensured with sealed opaque envelopes prepared by an independent researcher not involved in patient enrollment. This was a double-blind trial: patients and outcome assessors were blinded to treatment allocation, while nurses administering the anticoagulants were not involved in outcome evaluation.

### Interventions

*Enoxaparin group: received subcutaneous enoxaparin.

*Heparin group: received subcutaneous unfractionated heparin.

*Rivaroxaban group: received oral rivaroxaban.

All patients received perioperative care according to institutional protocols.

### Data Collection Tool

A structured clinical checklist was used to record demographic data, clinical variables, and outcomes. Content validity of the checklist was confirmed by three independent experts in gynecologic oncology. Reliability was assessed by inter-rater agreement in 10 pilot cases (>90% agreement). Cronbach’s alpha was not applied, as the checklist was not a multi-item psychometric scale.

### Study Variables and Definitions

Baseline variables: age, body mass index (BMI), employment status, cancer type, type of surgery, history of venous thromboembolism (VTE).

Primary outcome: intraoperative transfusion requirement (≥1 unit of packed red blood cells administered intraoperatively).

Secondary outcomes: dyspnea, chest pain, peripheral edema, lower limb pain, bleeding (WHO criteria), infection, hematoma, recovery, and mortality, assessed during hospitalization and at 1-week, 2-week, and 1-month follow-ups.

### Statistical Analysis

Data were analyzed using SPSS v.22 (IBM Corp., Armonk, NY, USA).

Normality of continuous variables was assessed with the Shapiro-Wilk test. Continuous variables were presented as mean ± standard deviation (SD) and compared using one-way ANOVA or Kruskal-Wallis test, as appropriate. Categorical variables were expressed as frequencies and percentages and compared using chi-square or Fisher’s exact test. Relative risks (RR) with 95% confidence intervals (CI) were calculated for key outcomes. A two-sided P<0.05 was considered statistically significant.

## Results

**Table:** Table1. Baseline Demographic Characteristics (Age, BMI)

**Variable**	**Enoxaparin (n=25)**	**Heparin (n=30)**	**Rivaroxaban (n=30)**	**P-value**
**Age (years), mean ± SD (Min-Max)**	57.4 ± 10.2 (41-74)	59.1 ± 9.6 (40-78)	58.6 ± 10.0 (43-76)	0.532
**BMI (kg/m²), mean ± SD (Min-Max)**	27.8 ± 4.9 (20.2-37.6)	28.5 ± 5.5 (19.4-39.2)	28.6 ± 5.2 (20.1-40.5)	0.056

Values are mean ± standard deviation (SD). Statistical test: ANOVA. Abbreviation: **BMI**=Body Mass Index.

**Table:** Table2. Baseline Clinical Characteristics (Cancer Type, Surgery Type, History of VTE)

**Variable**	**Enoxaparin (n=25)**	**Heparin (n=30)**	**Rivaroxaban (n=30)**	**P-value**
**Employment, n (%)**	5 (20.0)	6 (20.0)	5 (16.7)	0.910
**Staging hysterectomy, n (%)**	13 (52.0)	15 (50.0)	16 (53.3)	0.982
**Cytoreductive surgery, n (%)**	9 (36.0)	11 (36.7)	10 (33.3)	0.967
**Endometrial cancer, n (%)**	10 (40.0)	12 (40.0)	11 (36.7)	0.954
**Ovarian cancer, n (%)**	8 (32.0)	9 (30.0)	9 (30.0)	0.989
**Sarcoma, n (%)**	2 (8.0)	3 (10.0)	2 (6.7)	0.903
**History of VTE, n (%)**	2 (8.0)	3 (10.0)	2 (6.7)	0.903

Values are n (%). Statistical test: Chi-square test. Abbreviation: **VTE**=Venous Thromboembolism

**Table:** Table3. Intraoperative Transfusion Requirement

**Variable**	**Enoxaparin (n=25)**	**Heparin (n=30)**	**Rivaroxaban (n=30)**	**P-value**	**RR (95% CI)**
**Transfusion required, n (%)**	2 (8.0)	2 (6.7)	10 (33.3)	0.010	Riva vs Enoxa: 4.17 (0.95-18.2); Riva vs Heparin: 3.96 (0.91-17.3)

Values are n (%). Statistical test: Chi-square test. Abbreviations:**RR**=Relative Risk; **CI**=Confidence Interval

**Table:** Table4. Postoperative Complications (Discharge and Follow-ups)

**Complication**	**Time**	**Enoxaparin (n=25)**	**Heparin (n=30)**	**Rivaroxaban (n=30)**	**P-value**
**Dyspnea**	Discharge	1 (4.0)	1 (3.3)	2 (6.7)	0.812
**Chest pain**	Discharge	0	1 (3.3)	1 (3.3)	0.744
**Peripheral edema**	Discharge	0	0	1 (3.3)	0.367
**Peripheral edema**	1 week	0	1 (3.3)	5 (16.7)	0.046
**Lower limb pain**	1 week	1 (4.0)	1 (3.3)	2 (6.7)	0.873
**Bleeding**	2 weeks	0	1 (3.3)	1 (3.3)	0.744
**Dyspnea**	1 month	0	1 (3.3)	1 (3.3)	0.744

Values are n (%). Statistical test: Chi-square or Fisher’s exact test

**Table:** Table5-A. Late Postoperative Complications (Infection, Hematoma)

**Complication**	**Enoxaparin (n=25)**	**Heparin (n=30)**	**Rivaroxaban (n=30)**	**P-value**
**Infection, n (%)**	2 (8.0)	1 (3.3)	2 (6.7)	0.182
**Hematoma, n (%)**	1 (4.0)	1 (3.3)	2 (6.7)	0.182

Values are n (%). Statistical test: Fisher’s exact test

**Table:** Table5-B. Final Outcomes (Recovery, Mortality)

**Outcome**	**Enoxaparin (n=25)**	**Heparin (n=30)**	**Rivaroxaban (n=30)**	**P-value**
**Partial recovery, n (%)**	23 (92.0)	28 (93.3)	30 (100.0)	0.288
**Mortality, n (%)**	2 (8.0)	1 (3.3)	0	0.288

Values are n (%). Statistical test: Chi-square test.

**Table:** Table6. Frequency Distribution of Complications One Week, Two Weeks, and One Month after Discharge

				**One week after discharge **					
**Complication**					**Group**			**Chi-square**	**P value**
	**Enoxaparin**		**Heparin**		**Rivaroxaban**				
	**N **	**P**	**N **	**P**	**N **	**P**			
**Dyspnea**	No	25	100	29	96.7	30	100	1.738	<0.999
	Yes	0	0	1	3.3	0	0		
**Chest pain **	No	25	100	30	100	30	100		
	Yes	0	0	0	0	0	0		
**Peripheral edema**	No	25	100	29	96.7	25	83.3	5.450	0.046
	Yes	0	0	1	3.3	5	16.7		
**Lower limb pain**	No	25	100	29	96.7	27	90	3.079	0.322
	Yes	0	0	1	3.3	3	10		
**Bleeding**	No	24	96	29	96.7	29	96.7	0.464	<0.999
	Yes	1	4	1	3.3	1	3.3		
				**Two weeks after discharge **					
**Complication**					**Group**			**Chi-square**	**P value**
	**Enoxaparin**		**Heparin**		**Rivaroxaban**				
	**N **	**P**	**N **	**P**	**N **	**P**			
**Dyspnea**	No	25	100	29	96.7	30	100	1.738	<0.999
	Yes	0	0	1	3.3	0	0		
**Chest pain **	No	25	100	29	96.7	29	96.7	1.048	<0.999
	Yes	0	0	1	3.3	1	3.3		
**Peripheral edema**	No	25	100	28	93.3	25	83.3	4.666	0.099
	Yes	0	0	2	6.7	5	16.7		
**Lower limb pain**	No	25	100	29	96.7	28	93.3	1.554	0.772
	Yes	0	0	1	3.3	2	6.7		
**Bleeding**	No	24	96	30	100	28	93.3	2.502	0.328
	Yes	1	4	0	0	2	6.7		
				**One month after discharge **					
**Complication**					**Group**			**Chi-square**	**P-value**
	**Enoxaparin**		**Heparin**		**Rivaroxaban**				
	**N **	**P**	**N **	**P**	**N **	**P**			
**Dyspnea**	No	25	100	30	100	30	100		
	Yes	0	0	0	0	0	0		
**Chest pain **	No	25	100	30	100	30	100		
	Yes	0	0	0	0	0	0		
**Peripheral edema**	No	25	100	29	96.7	26	86.7	3.906	0.125
	Yes	0	0	1	3.3	4	13.3		
**Lower limb pain**	No	25	100	29	96.7	28	93.3	1.554	0.772
	Yes	0	0	1	3.3	2	6.7		
**Bleeding**	No	25	100	30	100	30	100		
	Yes	0	0	0	0	0	0		

**Table:** Table7. Frequency Distribution of Postoperative Complications

**Complication**			**Group**				**Chi-square**	**P-value**
	**Enoxaparin**		**Heparin**		**Rivaroxaban**			
	**N**	**P**	**N**	**P**	**N**	**P**		
**Infection**	2	8	0	0	0	0		
**Hematoma**	1	4	0	0	0	0		
**Pelvic hematoma **	0	0	1	3.3	0	0	8.466	0.182
**Infection** **and hematoma**	0	0	1	3.3	2	6.6		
**No complication **	22	88	28	93.4	28	93.4		
**Total**	25	100	30	100	30	100		

**Table:** Table8. Final Outcome of the Treatment Groups

**Postoperative complication**				**Group **			**Chi-square**	**P-value**
	**Enoxaparin**		**Heparin**		**Rivaroxaban**			
	**N**	**P**	**N**	**P**	**N**	**P**		
**No**	22	88	28	86.7	28	86.7	0.664	0.717
**Yes**	3	12	2	13.3	2	13.3		

**Table:** Table9. Final Outcome of the Treatment Groups

**Final outcome**				**Group **			**Chi-square**	**P-value**
	**Enoxaparin**		**Heparin**		**Rivaroxaban**			
	**N**	**P**	**N**	**P**	**N**	**P**		
**Partial recovery **	23	92	29	96.7	30	100	2.297	0.288
**Yes**	2	8	1	3.3	0	0		

This study was conducted on 85 patients undergoing gynaecologic oncology surgery. Baseline demographic and clinical characteristics of the patients are summarized in Table-1a and Table-1b. Table-1a shows continuous variables (age and BMI), while Table-1b presents categorical variables including employment status, type of surgery, cancer type, and history of vascular events, The mean age of the patients studied, enoxaparin, heparin, and rivaroxaban groups was 58.43±9.92, 58.12±12.28, 59.60±9.51, and 57.53±8.21 years, respectively. No significant difference was observed (Table-2) in terms of mean age between the groups (P=0.532). Also, the mean BMI of the patients studied was calculated to be 28.34±5.16 kg/m2, and no significant difference was observed in terms of BMI between the groups (P=0.056). The types of surgery performed included total hysterectomy and cytoreductive in 74 (87%), and 11 patients (13%), respectively. Also, the types of cancer in the patients studied included endometrial, cervical, ovarian, and sarcoma in 51 (60%), 8 (9.4%), 20 (23.5%), and 6 patients (7.1%), respectively. There was no significant difference between the treatment groups in terms of fre. Based on the results, 2 patients (3.4%) had a history of vascular events. In the enoxaparin group, none of the patients had a history of vascular events, and in the heparin and rivaroxaban groups, one patient had a history of vascular events. No significant difference was observed between the treatment groups in terms of history of vascular events (P=0.653). In terms of intraoperative complications, 14 patients (16.5%) required blood transfusion, of which 2 (8%), 2 (6.7%), and 10 patients (33.3%) in the enoxaparin, heparin, and the rivaroxaban groups required blood transfusion, respectively. The need for blood transfusion in the rivaroxaban group was significantly higher than in the other two groups (P≥0.05, Table-3). In terms of postoperative complications, dyspnea, chest pain, lower limb pain, and peripheral edema were reported in 1, 1, 4, and 2 patients in the heparin group, respectively. Also, lower limb pain was reported in 2 patients in the rivaroxaban group. No statistically significant difference was observed between the groups regarding postoperative complications (P≤0.05, Table-4). One week after the surgery, dyspnea was reported in 1 patient in the heparin group. Lower limb pain was observed in 1 and 3 patients in the heparin and rivaroxaban groups, respectively. Bleeding was observed in 3 patients, 1 in each (Table-5A, Table-5B) treatment group, and there was no statistically significant difference between the different groups in terms of the complications (P≤0.05, Table-6). This is while peripheral edema was observed in 1 and 5 patients in the heparin and rivaroxaban groups, respectively (P≥0.05).

Two weeks after discharge, dyspnea, chest pain, peripheral edema, and lower limb pain were observed in 1, 1, 2, and 1 patients in the heparin group, respectively. Also, chest pain, peripheral edema, lower limb pain, and bleeding were observed in 1, 5, 2, and 2 patients in the rivaroxaban group, respectively. In the enoxaparin group, no complications were reported, and no statistically significant difference was observed between the groups in terms of complications two weeks after discharge (P≤0.05). Also, one month after discharge, dyspnea, chest pain, and bleeding were not observed in any of the patients, but peripheral edema was seen in 5 patients (1 in the heparin and 4 in the rivaroxaban group). Also, lower limb pain was observed in 3 patients (1 in the heparin and 2 in the rivaroxaban group), but no significant difference was observed between two groups (P≤0.05). Infection and hematoma in the enoxaparin group, respectively. Also, pelvic hematoma and infection was observed in (Table-7) 1 patient in the heparin group, and infection and hematoma was observed in 2 patients in the rivaroxaban group. This is while no statistically significant difference was observed between the treatment groups (P≤0.05). In total, the complications were observed in 7 patients (3 in the enoxaparin, 2 in the heparin, and 2 in the rivaroxaban group), and there was no significant difference between the groups (P≤0.05, Table-8). The mortality rate in the enoxaparin and heparin groups was 2 (8%) and 1 (3.3%), respectively, and in the rivaroxaban group, all patients had partial recovery. There was no significant difference between the groups in terms of the mortality rate (P≤0.05, Table-9). In general, all three drugs studied were similar in terms of efficacy and safety, and no preference was observed in terms of thromboprophylaxis events.

## Discussion

In this pilot randomized controlled trial, we compared the effectiveness and safety of rivaroxaban, enoxaparin, and heparin for thromboprophylaxis in gynecologic oncology surgeries. The main findings were: (1) intraoperative transfusion requirements were significantly higher in the rivaroxaban group, with relative risk estimates 4-fold higher than enoxaparin and heparin; (2) peripheral edema was more common with rivaroxaban at one-week follow-up; (3) other short-term postoperative complications, including dyspnea, chest pain, lower limb pain, and bleeding, did not differ significantly between groups; and (4) long-term outcomes such as infection, hematoma, recovery, and mortality showed no statistically significant differences among groups.

Our results suggest that although rivaroxaban is widely used in other surgical and medical contexts, its application in gynecologic oncology surgeries may be associated with increased intraoperative bleeding risk, reflected by higher transfusion rates. This aligns with prior studies reporting variable bleeding profiles for direct oral anticoagulants compared to heparin-based regimens. However, the absence of significant differences in most postoperative complications and final outcomes suggests that rivaroxaban may still be a feasible alternative if bleeding risk is carefully managed.

Enoxaparin and heparin performed similarly across most outcomes. Both agents demonstrated lower transfusion rates and comparable safety profiles. The modest incidence of peripheral edema in the rivaroxaban group may reflect drug-specific pharmacodynamics, although this observation requires confirmation in larger cohorts.

The mortality rate, though low, occurred only in the heparin and enoxaparin groups, while no deaths were observed in the rivaroxaban arm. Given the small sample size, this finding should be interpreted with caution and not generalized. Importantly, the overall rate of partial recovery was high across all groups, indicating that all regimens were broadly effective for postoperative thromboprophylaxis.

### Strengths and Limitations

The strengths of this study include its randomized controlled design, double blinding, and prospective data collection on both intraoperative and postoperative outcomes. However, several limitations must be acknowledged. First, as a pilot study, the sample size was not powered to detect small differences between groups, limiting the generalizability of results. Second, unequal group sizes due to dropouts may have introduced imbalance despite randomization. Third, some outcomes were rare, reducing the ability to conduct robust statistical comparisons.

### Implications and Future Directions

Our findings highlight the need for caution in the use of rivaroxaban in gynecologic oncology surgeries, particularly regarding intraoperative bleeding risk. Larger, adequately powered multicenter RCTs are needed to confirm these results, refine risk stratification, and evaluate patient-centered outcomes such as quality of life and long-term thromboembolic events. Until such data are available, enoxaparin and heparin remain well-established options for perioperative thromboprophylaxis in this patient population.

## Conclusion

The results of our study indicated a greater need for blood transfusion in the rivaroxaban group than in the other two groups. However, no significant difference was observed between the groups in terms of discharge time, postoperative complications, and follow-up on the one week, two weeks, and one month after discharge. These results indicated the importance of thromboprophylaxis in gynaecologic oncology surgeries. Though initial research indicates that heparin, enoxaparin, and rivaroxaban might be equally safe and effective for thromboprophylaxis in gynaecologic oncology surgeries, these findings need to be verified. Additional large and multi-center randomized clinical trials are necessary to validate these findings and inform clinical practice.

## Conflict of Interest

The authors declare that they have no conflicts of interest.
